# Effects of Environmental and Water Quality Variables on Histamine-Producing Bacteria Concentration and Species in the Northern Gulf of Mexico

**DOI:** 10.1128/spectrum.04720-22

**Published:** 2023-06-13

**Authors:** Ashley Frith, Marlee Hayes-Mims, Ruth Carmichael, Kristin Björnsdóttir-Butler

**Affiliations:** a University of South Alabama, Mobile, Alabama, USA; b Dauphin Island Sea Lab, Dauphin Island, Alabama, USA; c U.S. Food and Drug Administration, Gulf Coast Seafood Laboratory, Dauphin Island, Alabama, USA; University of Minnesota Twin Cities

**Keywords:** histamine-producing bacteria, temperature, salinity, nutrients

## Abstract

Scombrotoxin (histamine) fish poisoning is a common seafood-borne illness attributed to toxin production by histamine-producing bacteria (HPB) in fish tissues during decomposition. In laboratory studies, growth of HPB and other bacterial species is affected by physical and chemical attributes, but natural communities of HPB are not well understood. To determine how *in situ* environmental and water quality variables may affect density of HPB in the natural aquatic environment, we compared presence and abundance of HPB to ambient temperature, salinity, dissolved oxygen, fecal coliforms, male-specific coliphage, nutrient concentrations, carbon and nitrogen stable isotope ratios, and C:N in water samples collected from July 2017 to February 2018 along a natural salinity gradient in a tidal river on the coast of northern Gulf of Mexico. HPB in water samples were quantified using a real-time PCR, most probable number method. HPB species were identified via 16S rRNA gene sequences. Temperature and salinity were determined to be the main factors driving HPB presence and concentration. Canonical correspondence analysis revealed that different HPB were associated with different environmental conditions. Photobacterium damselae was found under warmer, higher-salinity conditions; Raoultella planticola was found at colder, lower-salinity conditions; Enterobacter aerogenes was found at warmer, lower-salinity conditions; and Morganella morganii was found at most sites, independent of environmental conditions. These results showed that naturally occurring HPB abundance and species composition can be affected by environmental conditions, which could manifest in various potentials for histamine formation and scombrotoxin fish poisoning risk based on environmental factors.

**IMPORTANCE** This study determined the effects of environmental conditions on presence and abundance of naturally occurring histamine-producing bacteria in the northern Gulf of Mexico. Here, we show that HPB abundance and species composition are related to *in situ* ambient temperature and salinity, with the magnitude of this effect dependent on the particular HPB species. This finding suggests that environmental conditions at fishing sites could affect the risk of human illness from scombrotoxin (histamine) fish poisoning.

## INTRODUCTION

Scombrotoxin (histamine) fish poisoning (SFP) occurs worldwide (1) and is one of the most common seafood-borne illness in the United States (2, 3). Illness is caused by consumption of fish containing high concentrations of histamine (e.g., tuna, mahi-mahi, mackerel), leading to symptoms including nausea, diarrhea, skin rash, and a burning sensation in the mouth (4). Histamine is produced by histamine-producing bacteria (HPB), which use the histidine decarboxylase enzyme (HDC) to convert natural histidine in fish tissues to histamine as the fish decomposes (5). These bacteria consist of a variety of species (6) that can be present naturally in the environment and in the skin, gills, and gut of fish (7, 8). HPB can also be introduced after catch from fish handling and processing (8, 9). SFP is primarily attributed to time-temperature abuse, where harvested fish are stored at temperatures above refrigeration (4°C), allowing HPB to proliferate and produce histamine over time (10), although low-temperature histamine production is also possible (11, 12, 13).

Growth and activity of individual strains of HPB have been shown to vary with physical and chemical factors in laboratory settings. Increased temperature results in increased HPB growth, histamine production, and HDC activity for many species, including Morganella morganii, Morganella psychrotolerans, Photobacterium damselae, Photobacterium iliopiscarium, Photobacterium phosphoreum, and Raoultella planticola (13–20). Other factors, including salt content of growth media, oxygen availability, and pH are also known to affect growth, where HPB tend to increase with increased oxygen and decreased pH but salinity preferences vary by species (7, 14, 18–22). For example, cultured P. damselae grew at a slightly narrower range of conditions (15 to 35°C, 0.2 to 6% NaCl) than M. morganii (0.3 to 47°C, 0 to 6% NaCl) (7, 16). These studies of primarily single species in isolated cultures, however, do not address the complex suite of conditions that may affect HPB growth in the natural environment.

In the natural environment, HPB species must tolerate a broader combination of interacting environmental attributes and competitive microflora (23). HPB in the environment comprise a small percentage of the total bacterial community (24). It is possible that other unstudied variables, such as nutrients and the ratio of carbon to nitrogen (C:N) in the water, which affect conveyance and growth of other bacterial species, may indirectly affect the growth or species composition of HPB. Increased wastewater inputs and other nutrients generally result in increased bacterial load (25–27), and decreased C:N results in increased bacterial growth (28). These environmental variables can lead to shifts in bacterial species composition, even if individual conditions are within the tolerable range for the species. Furthermore, while changes in species composition have been documented for bacterial communities in general (29–32), studies have not specifically examined the effect of environmental factors on natural communities of HPB.

This study quantifies the effects of several *in situ* environmental and water quality attributes (temperature, salinity, pH, dissolved oxygen, nutrient concentrations) on the concentration and species composition of HPB along a salinity gradient in a tidal river. To determine if HPB presence and species composition are related to wastewater-associated nutrient and freshwater inputs in this location, we additionally tested for relationships to bacterial (fecal coliforms [FC]) and viral (male-specific coliphage [MSC]) indicators of human wastewater along with C and N stable isotope ratios in suspended particles at each site.

## RESULTS

### HPB concentrations and species.

HPB concentrations ranged from −1.13 to 4.38 log MPN 100 mL^−1^ (Table 1). Concentrations did not differ among sites (analysis of variance [ANOVA], *F*_4,13_ = 1.23, *P* = 0.35), but did differ among sampling months (ANOVA, *F*_3,14_ = 12.04, *P* < 0.001), with lower concentrations in January than in other months (Tukey’s *P* < 0.01 for all significant pairwise comparisons). A total of 390 *hdc*-positive bacteria were isolated from water samples, with the majority identified as M. morganii and *R. planticola* (Table 2). Fourteen other species were identified less frequently, each comprising <1% to 6% of species identified (Table 2).

**TABLE 1:** Measured HPB concentrations and corresponding environmental and water quality attributes at sampling sites

Site	Mo	HPB (log MPN 100 mL^−1^)	Temp (°C)	Salinity (PSU)	DO (mg liter^−1^)	pH	DIN (μM)	DON (μM)	TDN (μM)	FC (MPN 100 mL^−1^)	MSC (PFU 100 mL^−1^)	Mo	δ^13^C (‰)	δ^15^N (‰)	C:N
1	Jan	−1.04	8.9	27.4	8.92	8.31	4.91	23.03	27.94	<5	<10	Jan	−23.53	6.04	11.21
	Feb	1.38	16.1	13.0	8.03	7.85	17.17	21.74	38.91	265	<10	Feb	−23.57	4.89	6.92
2	Aug	3.63	30.7	15.8	4.39		0.87	26.90	27.77	<5	<10	Aug	−26.48	6.14	8.47
	Nov	2.97	23.7	18.2	5.63		0.58	26.68	27.26	10	<10	Nov	−27.11	6.87	7.21
	Jan	−1.13	9.7	25.7	10.05	8.37	0.18	18.00	18.18	10	<10	Jan	−23.92	6.57	13.94
	Feb	1.63	16.0	12.9	8.39	7.87	3.45	17.70	21.15	345	10	Feb	−23.95	5.43	8.71
3	Aug	3.38	30.6	15.7	4.37		1.71	28.97	30.68	5	<10	Aug	−26.48	6.15	8.59
	Nov	2.63	23.9	18.1	5.50		0.47	25.52	25.99	25	20	Nov	−27.83	6.16	6.24
	Jan	−0.42	9.8	25.7	10.08	8.37	0.44	18.54	18.98	15	<10	Jan	−23.95	6.86	11.43
	Feb	1.97	16.0	12.5	8.32	7.76	3.30	15.93	19.23	1,150	10	Feb	−23.32	4.67	9.34
4	Aug	3.38	32.6	8.6	5.39		1.54	45.42	46.96			Aug	−29.81	5.52	8.99
	Nov	2.97	22.8	8.4	4.56		7.77	31.69	39.46	55	20	Nov	−31.49	4.70	8.16
	Jan	0.97	9.0	9.7	9.93	7.77	2.69	27.93	30.62	80	<10	Jan	−26.99	5.64	8.45
	Feb	3.18	16.9	0.1	6.61	6.41	6.45	30.50	36.95	1,550	<10	Feb	−26.56	2.79	9.92
5	Aug	2.63	32.1	2.3	4.88		3.68	49.86	53.54	45	60	Aug	−32.01	5.66	9.86
	Nov	3.38	22.9	7.6	4.87		5.95	31.54	37.49	50	30	Nov	−31.18	5.53	8.82
	Jan	1.97	9.2	8.6	10.66	7.78	3.47	31.40	34.87	65	<10	Jan	−27.69	6.34	9.89
	Feb	3.30	16.8	0.0	6.42	5.24	9.84	40.50	50.34	1,300	10	Feb	−27.04	2.28	13.90

**TABLE 2:** *hdc*-positive species isolated from water samples

Species	% of isolates (*n* = 390)
Morganella morganii	48
Raoultella planticola	27
Photobacterium damselae	6
*Raoultella* spp.	3
*Morganella* spp.	2
*Aeromonas* spp.	1
*Citrobacter* spp.	1
Enterobacter aerogenes	1
Morganella psychrotolerans	1
Raoultella ornithinolytica	1
Pantoea dispersa	1
Plesiomonas shigelloides	1
Hafnia paralvei	<1
Klebsiella aerogenes	<1
Proteus *columbae*	<1
Pseudomonas spp.	<1

### Environmental attributes.

*In situ* environmental and water quality attributes varied temporally and spatially (Table 1). Water temperature did not differ among sites (ANOVA, *F*_4,13_ = 0.31, *P* = 0.87), but was different among months, where August was warmest and January coldest (ANOVA, *F*_3,14_ = 1,033, *P* < 0.001, Tukey’s *P* < 0.001 for all pairwise comparisons). Salinity was lower at site 5, farthest upstream in West Fowl River, than at sites 1, 2, or 3 (ANOVA, *F*_4,13_ = 6.09, *P* < 0.01, Tukey’s *P* < 0.05 for all significant pairwise comparisons). Salinity did not differ among months (ANOVA, *F*_3,14_ = 2.21, *P* = 0.13). Dissolved oxygen (DO) did not differ among sites (ANOVA, *F*_4,13_ = 0.22, *P* = 0.93) but was highest in January and lowest in August and November (ANOVA, *F*_3,14_ = 54.06, *P* < 0.001, Tukey’s *P* < 0.001 for all significant pairwise comparisons). pH did not differ among sites or dates for the period sampled (Jan to Feb) (ANOVA among sites: *F*_4,5_ = 1.16, *P* = 0.43; across dates: *F*_1,8_ = 4.18, *P* = 0.08).

Dissolved inorganic nitrogen (DIN) was higher at site 1, the site farthest west in Mississippi Sound, than at sites 2 or 3 near the river mouth (ANOVA, *F*_4,13_ = 3.89, *P* = 0.03, Tukey’s *P* < 0.05 for all significant pairwise comparisons), but did not differ among months (ANOVA, *F*_3,14_ = 2.69, *P* = 0.09). Dissolved organic nitrogen (DON) and total dissolved nitrogen (TDN) were higher at site 5 (upstream) than at sites 2 or 3 (ANOVA for DON, *F*_4,13_ = 4.75, *P* = 0.01, Tukey’s *P* < 0.05; for TDN, *F*_4,13_ = 6.89, *P* < 0.01, Tukey’s *P* < 0.01 for all significant pairwise comparisons) but did not differ among months (ANOVA for DON, *F*_3,14_ = 2.41, *P* = 0.11; for TDN, *F*_3,14_ = 1.29, *P* = 0.32).

FC concentrations did not differ among sites (ANOVA, *F*_4,12_ = 0.36, *P* = 0.83) but were higher in February than in other months (ANOVA, *F*_3,13_ = 8.93, *P* < 0.01, Tukey’s *P* < 0.01 for all significant pairwise comparisons). MSC levels did not differ among sites or months (ANOVA among sites, *F*_4,12_ = 1.34, *P* = 0.31; ANOVA among months, *F*_3,13_ = 1.75, *P* = 0.21).

δ^13^C values were higher at site 1 (downstream) than at site 4 (upstream) and were higher in February than in November (ANOVA among sites, *F*_4,13_ = 4.61; among months, *F*_3,14_ = 5.17, *P* < 0.05, Tukey’s *P* < 0.05 for both sets of comparisons). δ^15^N values did not differ among sites (ANOVA, *F*_4,13_ = 1.18, *P* = 0.36) but were lower in February than in all other months (ANOVA, *F*_3,14_ = 6.14, *P* < 0.01, Tukey’s *P* < 0.05 for all significant pairwise comparisons). C:N did not differ among sites or among months (ANOVA among sites, *F*_4,13_ = 0.41, *P* = 0.80; among months, *F*_3,14_ = 2.52, *P* = 0.10).

### Relationships between HPB and environmental attributes.

**FIG 1:**
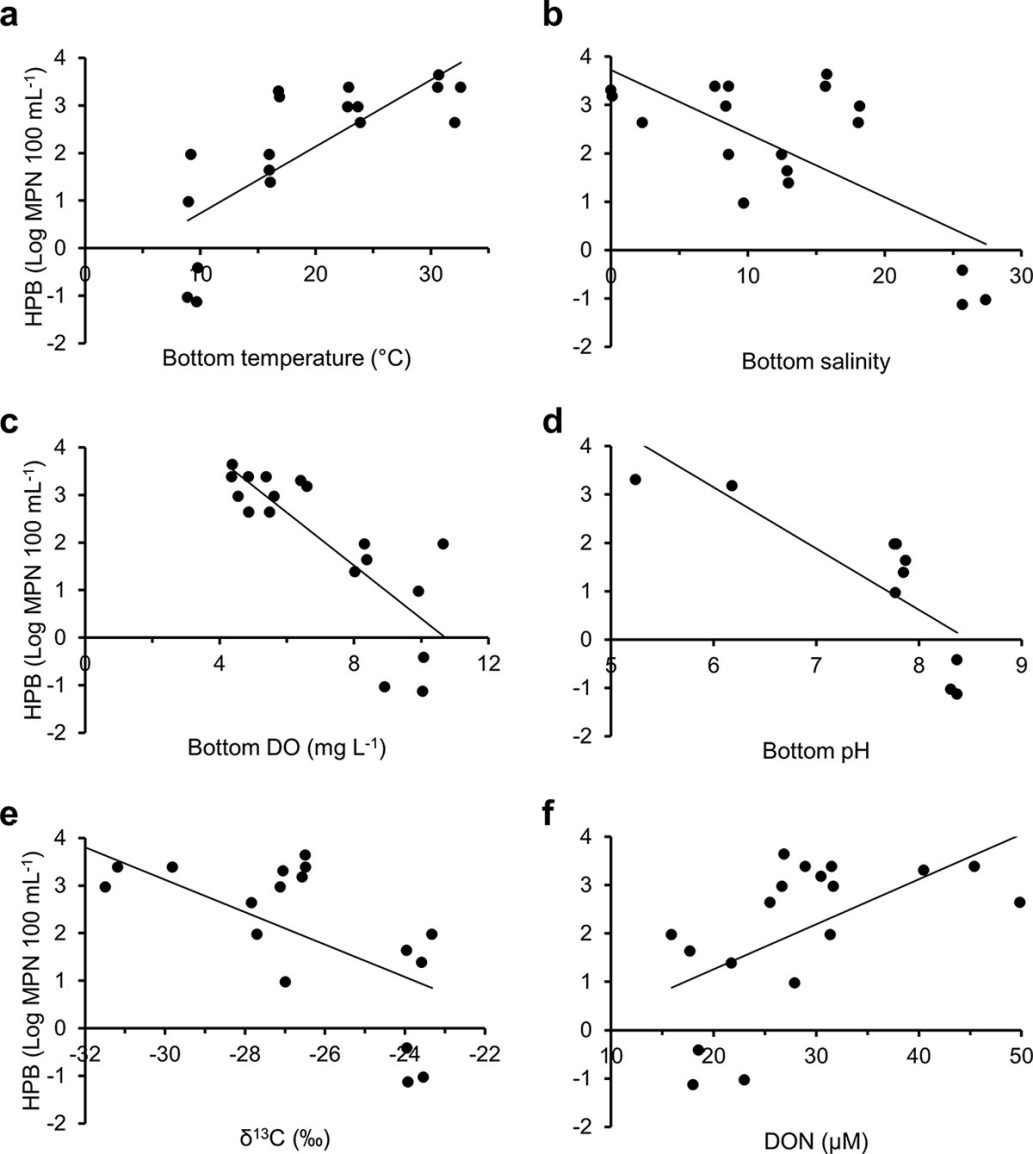
Significant relationships between HPB concentrations and environmental and water quality variables. Regression statistics are provided in Table 3.

Water temperature, salinity, DO, pH, δ^13^C, and DON had significant effects on HPB concentrations when analyzed individually, where HPB increased with increasing temperature and DON but decreased with increasing salinity, DO, pH, and δ^13^C (Fig. 1, Table 3). HPB were not significantly related to the other environmental attributes. Among the subset of environmental variables that were significantly related to HPB concentrations, all variables correlated with either temperature or salinity or both (see Table S1 in the supplemental material). When the individually significant factors were combined in a multivariable linear model, only temperature and salinity had significant effects on HPB concentration (Table 3). Specifically, in the multivariable model there was a 0.25-log increase in HPB for every 1°C increase in temperature and a 0.22-log decrease in HPB for every 1 unit increase in salinity (Table 3).

**TABLE 3:** Regression statistics for comparison of log-transformed HPB concentrations to individual environmental variables and to the significant variables in a multivariable model

y	*x*_1_	*x*_2_	Equation	*R* ^2^	df	*F*	*P*
HPB (log MPN 100 mL^−1^)	Temp (°C)		*y* = −0.66 + 0.14*x*	0.56	1, 16	22.40	<0.001
	Salinity (PSU)		*y *= 3.72 − 0.13*x*	0.49	1, 16	15.26	<0.01
	DO (mg/liter)		*y *= 5.98 − 0.56*x*	0.65	1, 16	30.07	<0.001
	pH		*y *= 10.76 − 1.27*x*	0.67	1, 8	16.10	<0.01
	δ^13^C (‰)		*y* = −7.09 − 0.34*x*	0.39	1, 16	10.36	<0.01
	DON (μM)		*y* = −0.61 + 0.09*x*	0.32	1, 16	7.52	0.01
	Temp (°C)	Salinity (PSU)	*y *= 1.13 + 0.11*x*_1_ − 0.10*x*_2_	0.82	2, 15	33.34	<0.001

**FIG 2:**
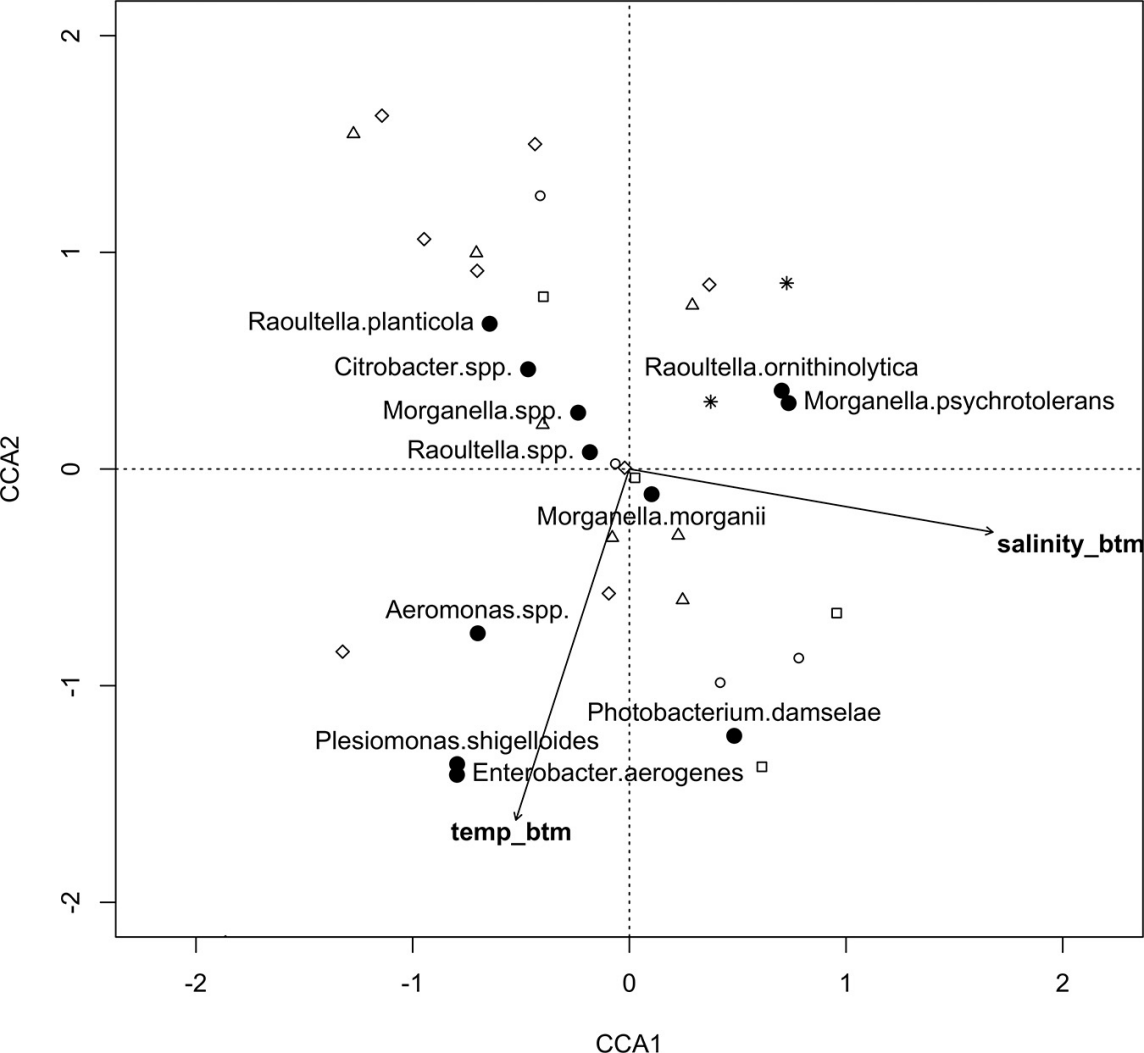
Canonical correspondence analysis (CCA) of the effects of environmental variables (arrows) on HPB species (filled circles). Sample collection sites are shown as open circles (site 1), open squares (site 2), open triangles (site 3), open diamonds (site 4), and stars (site 5). Only species comprising ≥1% of all isolates are plotted.

Presence of HPB species also varied primarily with salinity and temperature (*P*_model_ < 0.01, *P*_temperature_ < 0.01, *P*_salinity_ < 0.01) (Fig. 1), with responses differing among HPB species. Overall, CCA1 was a salinity gradient that explained 6.8% of the variation in HPB species composition (*P* < 0.01), and CCA2 was a temperature gradient that explained 5.4% of the species variation (*P* < 0.01) (Fig. 2). P. damselae and Pseudomonas spp. were mainly found at warmer, higher-salinity sites. *Morganella* spp., *Citrobacter* spp., and Raoultella planticola were found at colder, lower-salinity sites. Morganella psychrotolerans, Raoultella ornithinolytica, and Hafnia paralvei were found at colder, higher-salinity sites. *Aeromonas* spp., Plesiomonas shigelloides, Enterobacter aerogenes, Klebsiella aerogenes, and Proteus columbae were mainly found at warmer, lower-salinity sites. Morganella morganii and *Raoultella* spp. were found at many sampling points (53% and 25%, respectively) and were not strongly associated with either temperature or salinity, as shown by their proximity to the origin of the two axes (Fig. 2).

## DISCUSSION

The presence and concentrations of naturally occurring HPB in this study were primarily related to ambient temperature and salinity at sampling sites, and they varied spatially and seasonally depending on these variables. Overall, the combination of temperature and salinity explained about 80% of the variation in HPB concentrations and 12% of the variation in species composition. While changes in HPB species composition in relation to environmental factors have not been previously documented, an analysis of bacterial communities compiled from studies across a wide range of environments globally also found that salinity was the primary driver of species composition (33). Changes in species composition of aquatic bacteria (not specific to histamine producers) have been correlated with factors such as nitrogen, phosphorus, DO, and chlorophyll *a* in a variety of habitats (29–32, 34). Although DO concentration was highly related to HPB concentrations, the effect was opposite that of a previous study in which low DO inhibited bacterial growth (22). This inconsistency, combined with the strong negative correlation between temperature and DO and that DO was not a significant component of the multivariate models explaining HPB abundance or species composition, suggested that the relationship between HPB and DO was secondary to the temperature response in this case. Similarly, DON, δ^13^C, and pH also were related to HPB concentrations but not species composition. On the northern Gulf of Mexico coast, increased river discharge is typically associated with decreased salinity and conveys inorganic nutrients from land to water, decreasing δ^13^C (35) and increasing productivity and respiration, with potential for reduced pH (36). These relationships may explain the observed correlation between salinity and DON, δ^13^C, and pH and, in turn, apparent relationships to HPB concentration that were likely driven by salinity. The lack of relationships to HPB species composition, however, indicated that other variables, including those not measured in this study, may have contributed to species composition.

This study supports previous findings that the effects of environmental factors on the concentration of high histamine producers varies by HPB species. Some of this variation may be due to high tolerance for a range of temperature and salinity conditions in some species but not in others. For example, M. morganii was the most common species in this study and was not strongly associated with *in situ* temperature or salinity, in agreement with laboratory studies documenting the wide temperature and salinity tolerance of this species (7, 15, 18, 37). This tolerance may explain why M. morganii was common in this study, has been implicated in outbreaks of SFP worldwide, and is frequently mentioned in association with histamine production (18, 38, 39). In contrast, laboratory studies with *R. planticola* also documented a wide temperature tolerance (40, 41), but the closely related Raoultella ornithinolytica had a narrower salinity tolerance (42), which could explain why it was found at cold, fresh sites in this study. The *in situ* occurrences of Photobacterium damselae and Enterobacter aerogenes largely agreed with culture studies that determined the species’ optimum growth and histamine production were under warm and saline (7, 43) and under warm and fresh conditions (21, 44, 45), respectively. Additionally, Enterobacter aerogenes represented only 1% of the isolates in this study and similarly comprised only 2 to 11% of HPB isolated from swabs of Gulf of Mexico fish (7), suggesting that this high histamine producer is not dominant among the northern Gulf of Mexico marine bacterial communities. Although the specific conditions used for enriching and culturing bacteria in this study also could affect the HPB species composition, these results demonstrated that laboratory studies are generally consistent with field studies, suggesting that growth parameter data generated in laboratory studies may be used as predictive factors for natural HPB occurrence and abundance in the northern Gulf of Mexico, and perhaps elsewhere.

More in-depth studies are required to confirm whether similar factors drive HPB prevalence in the natural environment globally, especially in locations with different climate and salinity regimens. Future studies could benefit from using cultivation-independent methods to build upon the results from this paper. Regardless, because the concentration and species of HPB are related to environmental factors, it is possible that conditions at fishing locations will affect the risk of SFP. Although previous studies have not reported HPB concentrations in water samples, HPB concentrations in this study were 0.001 to 1% of total bacterial abundances estimated in other coastal systems (30, 46). This range is in agreement with a previous study which estimated that HPB comprise <0.1% of the total bacterial load on freshly caught fish (47). Although this proportion seems low, the fact that HPB in the environment remain sufficient to migrate to edible fish tissue and potentially cause human illness highlights the importance of understanding the factors that affect variation in naturally occurring HPB communities. Thus, additional sampling could help further identify the potential for SFP risk at times, in locations, or of fish caught from areas or seasons where conditions are most likely to result in higher concentrations of high histamine producers. This study also suggests that growth parameter data produced in laboratory studies may be predictive inputs for HPB prevalence in the natural environment and may be used to inform histamine and SFP control strategies. These results highlight that the ecology of naturally occurring HPB, which contributes to differences in HPB concentrations in freshly caught fish and subsequent histamine production during decomposition, may also contribute to management of SFP.

## MATERIALS AND METHODS

### Sampling.

**FIG 3:**
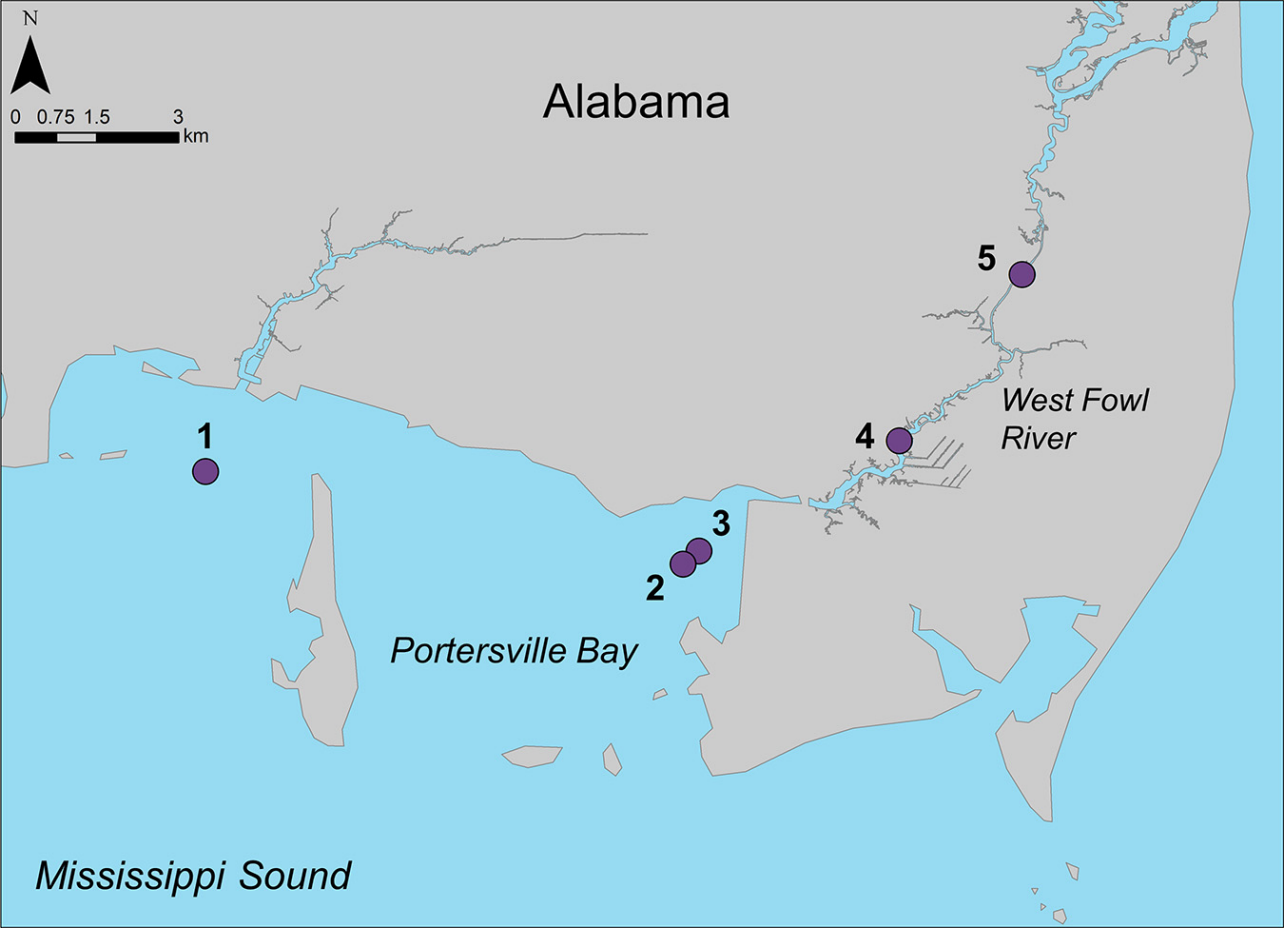
Sampling sites in Mississippi Sound and West Fowl River, Alabama.

To relate HPB to environmental and water quality variables, water samples and environmental data were collected at five sites along a natural salinity gradient (Fig. 3) from the Mississippi Sound into the upper reaches of West Fowl River, Alabama. Site 1 was located at the Bayou la Batre wastewater treatment plant outfall in Mississippi Sound; all other sites were at least 7.5 km away from the treatment facility (Fig. 3). Samples were collected four times between August 2017 and February 2018. Depth of sites ranged from approximately 0.9 to 2.2 m. Water samples for HPB quantification and identification were collected using a horizontal water sampler (Wildco, FL, USA) 0.1 m above the sediment surface in 4-liter sterile bottles (Nalgene, ThermoFisher Scientific, USA). To quantify FC and MSC, 500-mL surface water grab samples were collected in sterile bottles (Nalgene), and for stable isotope and nutrient analyses, two 1-liter mid-water-column samples were collected in acid-washed bottles (Nalgene) using the horizontal water sampler and prefiltered through 200-μm mesh. All bottles were stored upright in a plastic cooler with ice and processed within 24 h.

### HPB quantification.

To quantify HPB, water samples were serially diluted with 1× artificial seawater (12.2 g NaCl, 0.5 g KCl, 8.6 g MgSO_4_·7H_2_O, 1.0 g CaCl_2_·2H_2_O per 1 liter). One-liter, 100-mL, 10-mL, and 1-mL water samples and 10^−1^, 10^−2^, and 10^−3^ dilutions were enriched (1:10) with Luria 70% seawater broth containing 1% histidine (LSW-70BH; 350 mL 2× artificial seawater, 10 g tryptone, 5 g yeast extract, 10 g histidine per 1 liter) in triplicate for the 3-tube multiple dilution MPN series. Enrichments were grown at 30°C for 24 h, then 0.5-mL aliquots were taken from each enrichment, heated at 95°C for 10 min to release DNA, and frozen at −20°C until analysis. Boiled enrichments were centrifuged for 2 min at 12,000 × *g* immediately prior to analysis. A real-time PCR assay targeting the HPB was run on each enrichment dilution as described by Bjornsdottir-Butler et al. (48). Briefly, each 25-μL mixture contained 500 nM (each) forward and reverse primers, 300 nM probe, 5 mM MgCl_2_, 200 nM each deoxynucleoside triphosphate, 1× PCR amplification buffer, 2 μL internal amplification control DNA, and 2 μL DNA template. Cycling conditions were 95°C hold for 120 s, followed by 45 amplification cycles (94°C for 10 s, 58°C for 30 s, 60°C for 30 s). The MPN of HPB per 100 mL in each water sample was determined following the method of Blodgett (49), based on the number of dilutions and replicates identified as *hdc*-positive from the real-time PCR.

### HPB species composition.

To identify HPB, an aliquot was taken from the 1-liter enrichment (above), added 1:1 to deep-freeze medium (200 mL 1 M K_2_HPO_4_/NaH_2_PO_4_, 10 g yeast extract, 100 mL dimethyl sulfoxide, 100 mL glycerol per 1 liter), and stored at −80°C until analysis. Aliquots from samples stored in deep-freeze media were serially diluted with 1× artificial seawater and spread plated onto a Luria 70% seawater plate (LSW-70A [350 mL 2× artificial seawater, 10 g tryptone, 5 g yeast extract, 15 g agar per 100 mL]) and a violet red bile (VRBA) agar plate to encourage growth of marine and enteric bacteria, respectively. LSW-70A and VRBA plates were incubated at 30°C and 35°C, respectively, for 24 h. Forty-seven colonies were picked with sterile toothpicks from countable LSW-70A plates and inoculated into Luria 70% seawater broth (LSW-70B) in 96-well plates. Plates were incubated at 30°C for 24 h. Similarly, up to 47 pink colonies were picked from countable VRBA plates and grown in trypticase soy broth (TSB) in 96-well plates, which were incubated at 35°C for 24 h. Plates were heated at 95°C for 10 min in a thermal cycler and centrifuged at 2,235 × *g* for 10 min in a plate spinner. To identify HPB, an *hdc* real-time PCR assay was run on boiled cultures of each isolate following the methods of Bjornsdottir-Butler et al. (48). To screen isolates for 16S rRNA gene sequence identification, an additional species-specific real-time PCR targeting the common high-histamine producers M. morganii, Enterobacter aerogenes, Raoultella planticola*/ornithinolytica*, and P. damselae was run on each isolate following the methods of Bjornsdottir-Butler et al. (50).

Up to three of each *hdc*-positive/*hdc* species-specific-positive and all *hdc*-positive/*hdc* species-specific-negative isolates from each plate were moved forward for sequencing. To prepare for 16S rRNA gene sequencing, isolates were streaked for purity twice on LSW-70A or TSA plates according to initial growth conditions before inoculation into the corresponding LSW-70B or TSB. Inoculated cultures were incubated at 30°C for LSW-70B or 35°C for TSB for 24 h, then a 1.5-mL aliquot from each tube was centrifuged (12,000 × *g*, 2 min) and the supernatant was aspirated to obtain a bacterial pellet. DNA was purified on a QIAcube using a QIAamp DNA minikit (Qiagen) following the manufacturer’s instructions (QIAamp DNA Mini-Bacterial DNA kit), and the 16S rRNA gene was amplified using the universal primers Eubac 27F and Eubac 1492R as described by Delong (51). PCR products were cleaned using ExoSAP-IT PCR product cleanup reagent (Applied Biosystems, Waltham, MA) following the manufacturer instructions. Products were labeled for sequencing via PCR with the BigDye Terminator v3.1 cycle sequencing kit (Applied Biosystems) before cleanup with a Performa DTR V3 96-well short plate kit (Edge BioSystems, San Jose, CA), resuspension in Hi-Di formamide (Applied Biosystems), and Sanger sequencing on an ABI 3730xl DNA analyzer (Applied Biosystems), all following manufacturers’ instructions.

Sequencing reads were base-called with SeqA6 software (Applied Biosystems). Sequences were analyzed and compared to sequences in the NCBI 16S rRNA database using the BLAST algorithm in CLC Genomics Workbench software (Qiagen). HPB were identified to species level if maximum identification was at least 98%, otherwise to genus level, using an 16S rRNA NCBI database. If multiple species matched isolates with greater than 98% similarity, a neighbor-joining tree was created using an alignment with Jukes-Cantor measure of nucleotide distance and bootstrapping (100 replicates) using CLC Genomic Workbench software. The tree was used to discern between multiple species with >98% sequence similarities by assigning the identification of the nearest neighbor.

### Environmental attributes.

During each sample collection, a handheld YSI Pro 2030 (YSI Inc., Yellow Springs, OH) was used to measure the water temperature, salinity, and dissolved oxygen at 0.1 m above the sediment surface. During January and February, sampling was expanded to include use of a handheld YSI Professional Plus to measure the pH at 0.1 m above the sediment surface at each site.

For nutrient analyses, water samples were vacuum-filtered through preashed 0.7-μm glass fiber filters (MilliporeSigma), and the filters with particulate matter were reserved for stable isotope analyses. Filtrate was reserved in acid-washed bottles and stored at −20°C until analysis. Filtrate was analyzed on a San++ autoanalyzer (Skalar Analytical, NL) for NO_3_^−^ + NO_2_^−^, NO_2_^−^, NH_4_^+^, and TDN following established EPA colorimetric methods (52, 53, 54, 55) as described by Strickland and Parsons (56). Briefly, NO_3_^−^ + NO_2_^−^ was measured after copper-cadmium reduction to NO_2_^−^, and NO_2_^−^ was subtracted from the NO_3_^−^ + NO_2_^−^ complex (54, 56). NH_4_^+^ and TDN were measured using alkaline phenol and hypochlorite or persulfate oxidation methods, respectively (52, 53, 56). DIN was calculated as the sum of NO_3_^−^ + NO_2_^−^ + NH_4_^+^, and DON was calculated by subtracting DIN from TDN.

To quantify FC, 1 mL and 10 mL of each water sample were filtered through sterile 0.45-μm mixed cellulose esters filters (EZ-Pak) using a UV-sterilized vacuum filtration system (MilliporeSigma, Gelman Sciences) to capture FC. Filters were transferred onto mTEC (Acumedia) plates and incubated at 35°C for 2 h and then at 44.5°C for 24 h, after which time yellow, yellow-brown, or yellow-green CFU were counted.

A double-agar overlay method described by Cabelli (57) was used to quantify MSC, where 2.5 mL of each water sample was added to 2.5 mL soft agar tubes (tryptone, dextrose, NaCl, Difco agar, 1 M CaCl_2_ warmed in a 48.5°C water bath) along with 0.2 mL of the host strain (Escherichia coli Famp) grown in tryptone at 35°C for approximately 3 h. Tubes were mixed and plated on Famp agar plates (tryptone, dextrose, NaCl, Difco agar) and incubated for 24 h at 35°C before viral PFU were counted.

For stable isotope analyses, filters were dried to a constant weight in a 60°C oven (Fisher Scientific 3511FS gravity convection oven, Quincy Lab model 40 lab oven) and packed in tin capsules before sending to the UC Davis Stable Isotope Facility for δ^13^C and δ^15^N analyses by isotope ratio mass spectrometry using an Elementar Vario EL Cube or Micro Cube elemental analyzer (Elementar Analysensysteme GmbH, Hanau, Germany) interfaced to either an Isoprime VisION IRMS (Elementar UK Ltd., Cheadle, United Kingdom) or a PDZ Europa 20-20 isotope ratio mass spectrometer (Sercon Ltd., Cheshire, United Kingdom). Blank filters were run as a negative handling control. At least two replicate filters were analyzed for each site and sampling date, and one additional pseudoreplicate filter was analyzed following every 11 samples to quantify reproducibility in handling and analysis. The facility’s long-term instrument standard deviation is 0.2‰ for δ^13^C and 0.3‰ for δ^15^N, and the difference between pseudoreplicate pairs was 0.05‰ and 0.02‰ for δ^13^C and 0.20‰ and 0.02‰ for δ^15^N, well within the range of instrument error. The carbon and nitrogen content of each sample was used to calculate the molar C:N.

### Data analysis.

HPB, FC, and MSC concentrations were log-transformed prior to analysis. Nondetects are presented as “less than limit of detection” and were treated as zeros for statistical analyses. To determine how environmental attributes varied temporally and spatially, analyses of variance (ANOVAs) followed by *post hoc* Tukey’s test were used to analyze differences in attributes among sites and months (in the R version 3.5.0 “stats” package). To determine how HPB concentrations varied with environmental and water quality attributes, regression analyses (lm, in the R “stats” package) were used to compare HPB concentration to water temperature, salinity, DO, pH, DIN, DON, TDN, FC, MSC, δ^13^C, δ^15^N, and C:N. To account for colinear explanatory variables, significant variables from the individual regressions (except pH, due to the shorter sampling period) were combined in a multivariable linear model. Linear correlations among individually significant explanatory variables were tested using the Pearson’s product-moment correlation coefficient in R. Nonsignificant variables in the multivariable linear model were determined to be redundant due to the linear correlations and removed from the final model. To determine how environmental and water quality variables affected changes in HPB species composition, a canonical correspondence analysis (CCA) (58) was performed on species counts and environmental variables using the vegan package in R. Species counts were standardized using a log + 1 transformation for counts greater than zero, and zeros were left as zero (59). The significance of the CCA model, CCA axes, and explanatory variables were tested using 999 permutation tests with the anova.cca function in vegan. The CCA model was rerun with nonsignificant explanatory variables removed, and significance of the model, axes, and variables were retested using 999 permutation tests. Variables excluded from the CCA were DIN and DON, because they correlated with TDN, and pH, FC, and MSC, because of uneven sample sizes due to a shorter sampling period or sample loss in transport from the field to laboratory.

### Data availability.

The 16S rRNA gene sequences of HPB species are available in GenBank under accession numbers OQ878708 to OQ878958.
