# Supplementary material for: Effects of Environmental and Water Quality Variables on Histamine-Producing Bacteria Concentration and Species in the Northern Gulf of Mexico
Source: Microbiol Spectr. 2023 Jun 13;11(4):e04720-22. doi: 10.1128/spectrum.04720-22 (PMC10434188; doi:10.1128/spectrum.04720-22)
Supplement: Supplemental file 1 — Table S1. Download spectrum.04720-22-s0001.pdf, PDF file, 0.06 MB [file spectrum.04720-22-s0001.pdf]

Supplementary Information

Table S1: Significant correlations among the subset of environmental variables that significantly explained HPB concentrations.

x_1	x_2	r	p
Temperature	DO	-0.91	< 0.001
Temperature	pH	-0.63	0.05
Temperature	$\delta^{13}C$	-0.61	< 0.01
Temperature	DON	0.58	0.01
Salinity	pH	0.84	< 0.01
Salinity	$\delta^{13}C$	0.58	0.01
Salinity	DON	-0.68	< 0.01
DO	pH	0.80	< 0.01
DO	$\delta^{13}C$	0.64	< 0.01
DO	DON	-0.53	0.03
pH	DON	-0.81	< 0.01
$\delta^{13}C$	DON	-0.81	< 0.001
